# Author Response to Comment on: Conventional and Microwave Hydrothermal Synthesis and Application of Functional Materials: A Review

**DOI:** 10.3390/ma12213640

**Published:** 2019-11-05

**Authors:** Guijun Yang, Soo-Jin Park

**Affiliations:** Department of Chemistry, Inha University, 100 Inharo, Incheon 402-751, Korea; yanggj91@gmail.com

**Keywords:** crystal nucleation, crystal growth, temperature region, hydrothermal method

## Abstract

We recently published an article “Conventional and Microwave Hydrothermal Synthesis and Application of Functional Materials: A Review” on Materials, and we are honored to receive a comment article from Jalouli et al. We will give a detailed explanation for the confusion of the mechanism of crystal nucleation and growth in the comment article.

This short comment article is a response to Jalouli et al. and their misunderstanding of the mechanism of my recently published article [1]. Firstly, thank you very much for making such a comment on my article and giving me the opportunity to discuss academic issues with you. The problem that Jalouli et al. referred to is the second step in the mechanism of the hydrothermal reaction process, crystal nucleation and growth.

(1) The so-called hydrothermal method relies on the solubility of minerals in high pressures and high temperatures. Please note that this is under high temperature and high pressure.

As Jalouli et al. said, “The solubility reduction caused by the decrease in the dielectric constant of water at high temperatures is reduced.” This is commonsense and absolutely correct. However, high temperatures and high pressures not only reduce the dielectric constant of the solution significantly, but they also reduce the viscosity of the solution, which causes ion migration to be intensified, and the influence of the latter can offset the former.

In addition, Jalouli et al. declared that “this supersaturation acts as the driving force of nucleation and growth and results in the nanoparticles precipitation [2]”, which is not comprehensive. This is just one of many factors. Please read the article you cited carefully. The focus of this paper is to study the kinetic mechanism of solution nucleation. Under non-hydrothermal conditions, this is completely established and correct, but under hydrothermal conditions, the mechanism changes. It is certain that supersaturation causes nucleation to be a factor, but it is not the main factor any more.

As stated herein, “the crystal growth is performed in an apparatus consisting of a steel pressure vessel called an autoclave, in which a nutrient is supplied along with water. A temperature gradient is maintained between the opposite ends of the growth chamber. At the hotter end the nutrient solute dissolves, while at the cooler end it is deposited on a seed crystal, growing the desired crystal” [3]. I am sure you can find this sentence easily on Wikipedia. What is emphasized here is nucleation in the low temperature region, rather than nucleation at low temperatures. Nucleation occurs due to the presence of a temperature gradient. This is also the definition of the hydrothermal method. In addition, what is highlighted here is the existence of a temperature gradient, rather than simply considering the mechanism of low or high temperatures. I think here, you might have a misunderstanding of the low/high temperature region and the low/high temperature. 

In addition, it should be added that the thermal diffusion coefficient of the solution under hydrothermal conditions is larger than that of normal temperatures and normal pressures. This indicates that the hydrothermal solution has a greater convective driving force, which is beneficial to crystal nucleation and growth.

(2) Jalouli et al. claimed that “high temperatures are more conducive to nucleation and growth”. This is also not comprehensive. As described above, in the whole process of crystal growth, both high temperatures and low temperatures are relatively terms, but the temperature difference is always present. The temperature difference causes convection, the solute in the high temperature region dissolves, and the seed crystal grows in the low temperature region.

In general, why should the heating temperature rise slowly from room temperature to the target temperature at a certain heating rate during the experiment? This is uniform crystal nucleation and growth within a certain temperature gradient. According to what Jalouli et al. claimed, simply, the high temperature is conducive to the reaction, therefore, we can completely configure the solution at a high temperature and put it into the reaction vessel to directly react at the target high temperature. If we do it like this, will the crystals get more perfect? Of course, they will not—I just made an extreme hypothesis to illustrate the problem.

Thanks again for your comment on the article. If you have any questions, please feel free to discuss with me or the editor’s office. 

## References

[1] Yang G., Park S.-J. (2019). Conventional and microwave hydrothermal synthesis and application of functional materials: A review. Materials.

[2] Nývlt J. (1968). Kinetics of nucleation in solutions. J. Cryst. Growth.

[3] Hydrothermal synthesis—Wikipedia. https://en.wikipedia.org/wiki/Hydrothermal_synthesis.

